# Spontaneous Complete Regression of Colon Cancer Liver Metastases in a Lung Transplant Patient: A Case Report

**DOI:** 10.1155/2023/9643370

**Published:** 2023-01-12

**Authors:** Koen Zwart, Dieuwertje Ruigrok, Magda de Graaf-Bos, Roel Goldschmeding, Miriam Koopman, Guus M. Bol

**Affiliations:** ^1^Department of Medical Oncology, Imaging & Oncology, University Medical Centre Utrecht, Utrecht, Netherlands; ^2^Department of Pulmonology, University Medical Centre Utrecht, Utrecht, Netherlands; ^3^Department of Pathology, University Medical Centre Utrecht, Utrecht, Netherlands

## Abstract

**Background:**

Cancer has become an important cause of death in solid organ transplant patients. The cause of malignancies in patients with solid organ transplants is multifactorial, but the use of intensive immunosuppression is regarded as an important factor. We describe the spontaneous, complete regression of colon cancer liver metastases, without initiation of antitumor therapy, in a solid organ transplant patient after modulation of immunosuppressants. *Case Presentation.* A 59-year-old female was admitted with fever, general discomfort, and elevated liver enzymes. She had received a single lung transplant, five years prior, for end-stage chronic obstructive pulmonary disease. Abdominal ultrasound and a computed tomography scan showed extensive liver lesions, and liver biopsy determined that the lesions were liver metastases originating from a colonic adenocarcinoma. Histopathologic analysis revealed that the primary tumor and liver metastases were mismatch repair-deficient (BRAF^V600E^ mutant and MLH1/PMS2-deficient), also known as a microsatellite instable tumor. The patient's clinical condition deteriorated rapidly, and she was discharged home with palliative care. No antitumor treatment was initiated. Additionally, there was a short period without any immunosuppressants. Unexpectedly, her clinical condition improved, and complete regression of liver metastases was observed on imaging two months later. Unfortunately, the patient developed rejection of her lung transplant and succumbed to pulmonary disease six months following her cancer diagnosis. The autopsy confirmed the primary colon tumor location and complete regression of >40 liver metastases.

**Conclusions:**

Disinhibition and reset of the host immune response could have led to immune destruction of the liver metastases of this patient's immunogenic dMMR colon carcinoma. This case underscores the huge impact that temporary relief from immunosuppressive therapy could have on tumor homeostasis. Balanced management of care for organ transplant recipients with malignancies requires a multidisciplinary approach involving medical oncologists and transplant physicians to reach the best quality of care in these complex cases.

## 1. Introduction

Over the past decades, survival of patients with solid organ transplants has increased through developments in surgical techniques and improvements of immunosuppressants [1]. Malignancies present two to three times more often in posttransplant patients compared to the general population and are becoming an important factor in the survival of posttransplant patients [2]. With the longer lifespan, malignancies are one of the major causes of death for posttransplant patients [3].

The cause of malignancies in patients with solid organ transplants is multifactorial. However, the use of intensive immunosuppression is regarded as an important factor [4, 5]. Immunosuppression allows the proliferation of oncogenic viruses and diminishes cancer cell immune surveillance [5, 6]. Immunogenic cancers like skin cancer, Kaposi's sarcoma, and posttransplant lymphoma are well-known cancers associated with solid organ transplantation. Posttransplant patients also have a higher incidence of colorectal cancer (CRC), although the underlying mechanism of this association is unknown [2, 7, 8].

We report the case of a patient with metastatic colon cancer, deficient in its mismatch repair system (dMMR), also known as a microsatellite instable (MSI) tumor. This subset of CRC is considered highly immunogenic [9]. It can arise by the hereditary nonpolyposis colon cancer syndrome, better known as the Lynch syndrome, or sporadically. In our case, the tumor harbored a BRAF^V600^ mutation, which makes it almost certain that the dMMR tumor originated through a sporadical pathway [10]. The prevalence of dMMR in metastatic CRC is approximately 5% [11], but might be higher in posttransplant patients under immunosuppression. Literature regarding this topic is scarce. In one study, four out of 12 posttransplant patients with CRC were dMMR [12]. The patient we present here remarkably showed complete regression of massive tumor metastases in the liver upon only short relief from immunosuppression.

## 2. Case Presentation

A 59-year-old woman was presented to the hospital with fever and general discomfort. She had a unilateral left-sided lung transplantation five years prior because of end-stage chronic obstructive pulmonary disease. The lung transplantation was complicated over the past years by recurrent infections and cytomegalovirus (CMV) reactivations under systemic immunosuppressive therapy. She presented with iron-deficiency anemia two years prior. A colonoscopy was recommended but declined by the patient. A fecal occult blood test was obtained, which turned out to be negative at the time. She was admitted to the hospital four times over the last two years because of infectious episodes. The patient's routine medication included, among others, prednisolone 10 mg, mycophenolic acid 360 mg twice daily, tacrolimus (target trough level 8 mcg/L), acenocoumarol, and ferrous fumarate 200 mg. On examination, she had a blood pressure of 100/50 mmHg, a heart rate of 90 beats per minute, body temperature of 39.1 degrees Celsius, audible fine crackles in the right lower lung fields, and a 98% peripheral oxygen saturation without supplemental oxygen. Further physical examination was unremarkable.

Blood analysis showed C reactive protein of 124 mg/L (normal range: 0-10 mg/L), hemoglobin of 6.8 mmol/L (normal range: 7.4-9.6 mmol/L), creatinine of 197 *μ*mol/L (normal range: 49-90 *μ*mol/L), and elevated liver enzymes, including an increase in alkaline phosphatase of 182 U/L (normal range: 0-120 U/L) and gamma-glutamyl transferase of 281 U/L (normal range: 0-40 U/L). A throat swab, CMV viral load, and Epstein-Barr virus (EBV) viral load were negative. Urine sediment showed 250 leukocytes/*μ*L and urine culture was positive for *E. coli*. She was diagnosed with a complicated urinary tract infection and was treated with intravenous antibiotics, from which she slowly recovered.

Because of unexplained elevated liver enzymes and an increased level of creatinine, an ultrasound of the abdomen was made, which revealed lesions suspicious for liver metastases. Positron emission tomography-computed tomography (PET-CT) showed focal high FDG uptake in the right colon and more than forty different liver lesions. The maximum standardized uptake value (with a bodyweight corrected formulation) of the liver lesions was 14.6. Subsequent liver biopsy confirmed the diagnosis of colon cancer liver metastases. Immunohistochemical analysis showed dMMR, by loss of expression in MLH1, PMS2, and MSH6. Molecular analysis found a BRAF^V600E^ mutation, with no mutation in HRAS, KRAS, and NRAS. In our case, dMMR probably developed sporadically, since a BRAF^V600E^ mutation is strongly correlated with a sporadical originating pathway [10].

**Figure 1:**
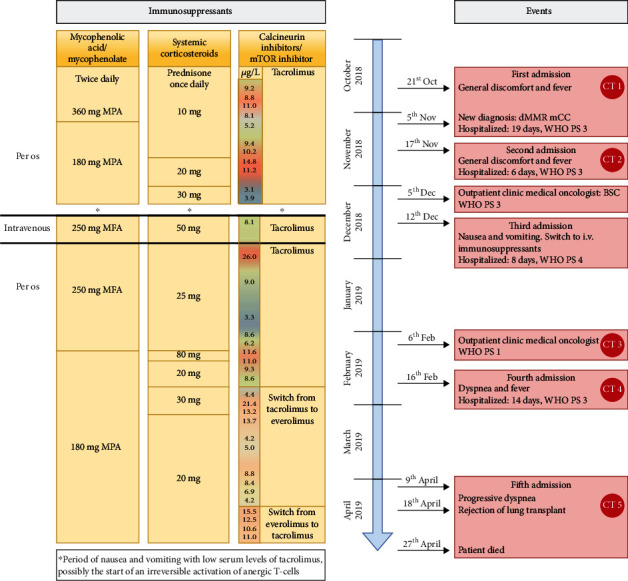
Timeline with dose of immunosuppressants and events. Target trough levels of tacrolimus and everolimus were 7-8 *μ*g/L and 6-8 *μ*g/L, respectively, with blue coloring for subtherapeutic levels, green for therapeutic levels, and red for supratherapeutic levels. Abbreviations: dMMR = deficient mismatch repair; i.v. = intravenous; mCC = metastatic colon cancer; MFA = mycophenolate; MPA = mycophenolic acid; MSI = microsatellite instability; mTOR = mechanistic target of rapamycin; WHO PS = World Health Organization performance score.

After recovering from a second hospitalization due to general discomfort and fever, the patient was seen in the outpatient clinic by a medical oncologist to discuss the current oncologic treatment options. Unfortunately, the patient deteriorated before any antitumor treatment could be initiated. Patient management was focused on best supportive care, and nonessential medications were discontinued. Prednisone was increased for symptom relief, and other immunosuppressants were continued.  Figure 1 presents a timeline with dose of immunosuppressant and events. Repeated CT imaging showed substantial progression of disease since diagnosis.

Shortly thereafter, the patient was admitted to the hospital due to nausea and vomiting lasting for seven until ten days, which made her unable to take any oral medication. This led to a serum tacrolimus trough level of 3.1 *μ*g/L, which is well below the target level of 7-8 *μ*g/L. The patient temporarily switched to intravenous immunosuppressive therapy and could be discharged after normalization of tacrolimus levels and improved control of nausea and vomiting complaints. The prognosis at this point was estimated to be a few weeks at most, and ambulatory best supportive care was continued.

**Figure 2:**
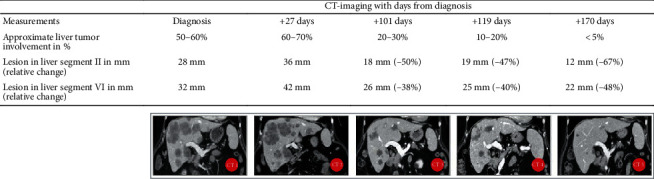
CT-imaging with measurement of lesions in liver segments II and VI and approximate liver tumor involvement. Abbreviations: CT = computerized tomography.

The patient contacted her medical oncologist after two months for reevaluation of her condition. Her performance status had unexpectedly improved, and CT imaging showed reduction of the colon cancer liver metastases (Figure 2). Since the tumor already shrunk significantly, no further antitumor treatment was initiated, and periodic follow-up was planned at the outpatient clinic of the medical oncologist. After interdisciplinary deliberation with her treating pulmonologist and medical oncologist, tacrolimus was switched to everolimus because of the possible antitumor effects of mammalian target of rapamycin (mTOR) inhibitors [13] and possible deleterious effect of calcineurin inhibitors on tumor progression [14, 15].

Shortly afterwards, the patient was admitted because of dyspnea and fever due to a mycoplasma pneumoniae and aspergillosis, from which she recovered after receiving antibiotics, increased dosage of corticosteroids, and antifungal (azole) treatment. At a follow-up appointment in the outpatient clinic, the pulmonologist reinitiated a proton-pump inhibitor, pneumocystis pneumonia prophylaxis, and CMV immunoglobulins. However, her dyspnea progressed and lung function significantly declined, leading to readmission. Unfortunately, her condition deteriorated rapidly, and total lung capacity measurement was not possible anymore. A high-resolution CT showed signs of restrictive allograft syndrome (RAS). A conventional CT demonstrated further regression of colon cancer liver metastases. Despite initiating treatment with azithromycin, montelukast, and broad-spectrum empiric antibiotics, progressive respiratory insufficiency developed. Morphine was given for symptom relief, and ultimately, palliative sedation was started, shortly after which she died.

**Figure 3:**
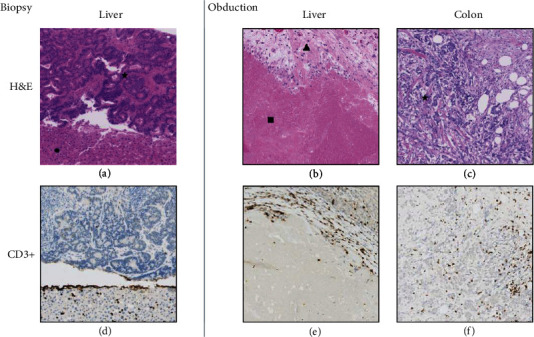
Histopathology of primary tumor and liver metastases. H&E staining of liver and colon sections (a–c). Liver biopsy sections with an asterisk showing colorectal metastasis and a circle showing preexistent liver tissue (a), liver obduction sections with a square showing necrosis and a triangle showing fibrosis (b), and colon carcinoma obduction sections with an asterisk showing colon tumor tissue (c). CD3+ staining of liver and colon sections (d–f). Liver biopsy sections, liver obduction sections, and colon obduction sections all show a sparse lymphocytic T-cell infiltrate. Magnification of (a), (b), (d), and (e) is 25x and (c) and (f) is 20x.

Before the patient deceased, she and her family consented to an autopsy. Remarkably, the autopsy confirmed complete regression of >40 colon cancer liver metastases. However, no regression of the primary mucinous adenocarcinoma of the caecum seemed to have occurred. The primary tumor showed loss of expression in MLH1 and PMS2 and, in contrast with the findings of the liver metastases, no loss of expression in MSH6 was observed. Lymphocytic infiltrate was scarce in both the primary tumor as in the necrotic/fibrotic liver lesions with some CD3+ cells, a slight number of CD8+ cells, and only a few positive cells with granzyme B staining (Figure 3). In the necrotic lever lesions, no vital tumor cells were present (Figure 3).

The lung transplant showed histopathological signs of bronchiolitis obliterans syndrome and diffuse alveolar damage. These pathological features in combination with clinical reduction of the forced expiratory volume in one second (FEV1) from baseline and persistent specific aberrations on high-resolution CT imaging are consistent with chronic lung allograft dysfunction based on a mixed phenotype of bronchiolitis obliterans syndrome and RAS, where the RAS component likely explains the very rapid decline.

## 3. Discussion

International clinical guidelines recommend immunotherapy for dMMR mCRC patients [16]. The 24-month overall survival was 48% in the pembrolizumab arm versus 19% in the chemotherapy arm [17]. However, patients with solid organ transplants were not included in these large trials. A retrospective pooled study of 39 patients reviewed the administration of immune checkpoint inhibitors in solid organ transplant cancer patients and observed graft loss in 81% of patients, with a median time to rejection of 16 days and a mortality rate of 46% [18]. Therefore, these patients often forego treatment with immune checkpoint inhibition.

Spontaneous regression of a tumor is rare, the cause is often not known, and most hypotheses are built around the role of inflammation, infection, and the immune system [19]. Most cases with spontaneous regression of metastases occur in immunogenic tumors like renal cell carcinoma, neuroblastoma, and malignant melanoma [19]. Possible spontaneous regression has also been reported in metastatic CRC in the period 1927-2005 [20]. However, this has never been documented as detailed and extensively as for the current case.

We have considered several hypotheses on how complete spontaneous regression of the colorectal liver metastases in this patient occurred. Our main hypothesis is that the immune system played an important role in the regression of the metastases [21]. The frame shift mutation rate in dMMR CRC tumors is hundredfold higher than in proficient mismatch repair CRC tumors, which results in a high number of immunogenic neoantigens [22]. When these neoantigens are presented by an antigen-presenting cell and subsequently recognized by T cells, an immune antitumor response can occur [23]. However, when on immunosuppressants, this immune antitumor response is severely impaired or even completely afunctional [24]. Our patient experienced a period of severe nausea and was unable to use her immunosuppressive medications (Figure 1, see asterisk), causing temporary subtherapeutic levels of immunosuppressants. During this short period in which the immune system was uninhibited, anergic T cells could possibly have been irreversibly activated. These activated T cells might have subsequently led to an antitumor response, which continued despite serum level of immunosuppressants returning to normal. However, we did not observe a strong immune reaction by immunohistochemistry at the time of obduction, with limited CD3+ (Figure 3) and CD8+ cells without any granzyme B staining. A reason for the lack of immunohistochemical proof of an immune response could have been the complete regression of the colorectal liver metastases, with an already extinguished immunological response when obduction ensued. Interestingly, while the liver metastases completely regressed, the primary tumor did not. With the unexpected additional loss of expression in MSH6 in the liver metastases, in contrast to the primary tumor, the liver metastases could have contained more immunogenic neoantigens than the primary tumor. Intratumoral heterogeneity, with an additional loss of expression in MSH6 in the liver metastases, can be the result of the manner of subclonal immune selection in dMMR tumors [25].

Alternatively, a metabolic catastrophe could have led to the complete regression of the liver metastases. Additional mutations in the liver metastases might have caused unconditional activation of cell growth with rapid proliferation and progression, as was also observed between the first and second CT scan, possibly leading to severe nutritional deprivation, hypoxia, and metabolic stress, triggering autophagy [6, 26, 27]. After the third admission, our patient suffered from severe anorexia, and she did not have any intake for almost three weeks. Perhaps, this nutritionally deprived state added to the metabolic stress in the liver metastases. The most important argument against this hypothesis is the fact that many late-stage cancer patients have periods of severe anorexia, which does not lead to a survival benefit but instead the contrary.

Another explanation could be tumor infarction due to vascular thrombosis in the tumor microenvironment, but there was no histopathological evidence for this at autopsy. Also, the possible substitution of everolimus, which was substituted for tacrolimus due to potential antitumor effects, is unlikely to have been the primary cause of complete regression, since CT imaging already showed rapidly regressing liver metastases before the switch in medication was made [13].

Under our main hypothesis, which places an important role of the immune system in regression of the metastases, a difficult dilemma arises in solid organ transplant recipients developing immunogenic tumors. Based on the etiology of immunogenic tumors, and as described in literature, one might try to keep immunosuppressive levels as low as possible to have a positive impact on the tumor [5]. On the other hand, when lowering immunosuppression, the chance of rejection of the allograft increases. A possible solution could be to temporarily lower immunosuppression, causing an irreversible immune response against the tumor, whereafter immunosuppression levels can be adjusted to normal again. However, the balance of immunosuppression in reducing the tumor load while preventing rejection is a complex conundrum in which an interdisciplinary approach is crucial.

In our case, we show the exceptional, complete spontaneous regression of tumor metastases. To the best of our knowledge, this is the first time that this is described for colorectal liver metastases in a solid organ transplant patient without initiation of antitumor therapy. The immune system has likely played an important role in this case. Rejection of the patient's lung transplant emphasizes the delicate balance of lowering immunosuppressive therapy to activate the anticancer immune response versus effective immunosuppressive therapy to prevent rejection. This emphasizes the need for interdisciplinary collaboration between medical oncologists and transplant physicians to reach the best quality of care in these complex cases and could improve understanding in the complex relationship of immunosuppression treatment and immunogenic tumors.

## 4. Next of Kin Perspective

Son: “my father called me on my cell phone to tell me there was something wrong with my mother, although the severity of it was not directly apparent. There was a small chance it would be cancer. Two days later, I received another telephone call from my father. He told me everything was completely amiss, cancer was diagnosed, and it was everywhere.”

Father: “the diagnosis of cancer was a slap in the face. We were heartbroken. Not much later, when my wife was back home, there was a point in time she had not eaten in three weeks. We knew she was very sick, and the doctors had told us she was dying. One evening, there was such a setback. We thought she was going to die that same night. The next day, quite suddenly, she started eating again. She was feeling a little bit better every day, and a month later, she was in a wheelchair in the local bar! It was almost incomprehensible. When we came back to the oncologist, we could not believe the cancer had shrunk, but the CT scan clearly showed as much! It was truly not comprehensible. The medical oncologist even told us that he had not experienced such a case ever before. It felt like a miracle. There was a lot of happiness, but unfortunately, it only lasted around six weeks. After that period, there were clear signs of lung rejection, and after the hope we had gotten, the realization of a very poor prognosis again was extra painful. We knew the dosing of the immunosuppressants must have been hard for the doctors. We were happy with every day we had won, although in hindsight I sometimes think: ‘could we have spared her something?' We wanted to fight, and with her relatively young age, the doctors agreed to continue treatment as long as they could. We trusted the doctors very much. They clearly communicated a lot with each other as well and always were on the same page.”

Son: “my mother always told me that the person who donated the lung to her had given her a second life. If she could donate her body to education and science, and help others with it, the circle would be complete again.”

## Data Availability

All data generated or analyzed during this study are included in this published article.

## Abbreviations

BSC: Best supportive care
CMV: Cytomegalovirus
CRC: Colorectal cancer
CT: Computed tomography
dMMR: Deficient mismatch repair
EBV: Epstein-Barr virus
IV: Intravenous
mCC: Metastatic colon cancer
mCRC: Metastatic colorectal cancer
MFA: Mycophenolate
MPA: Mycophenolic acid
MSI: Microsatellite instable
mTOR: Mechanistic target of rapamycin
PET-CT: Positron emission tomography-computed tomography
RAS: Restrictive allograft syndrome
WHO PS: World Health Organization performance score.

## References

[1] Lodhi S. A., Lamb K. E., Meier-Kriesche H. U. (2011). Solid organ allograft survival improvement in the United States: the long-term does not mirror the dramatic short-term success. *American Journal of Transplantation*.

[2] Engels E. A., Pfeiffer R. M., Fraumeni J. F. (2011). Spectrum of cancer risk among US solid organ transplant recipients. *JAMA*.

[3] Hall E. C., Pfeiffer R. M., Segev D. L., Engels E. A. (2013). Cumulative incidence of cancer after solid organ transplantation. *Cancer*.

[4] Vajdic C. M., van Leeuwen M. T. (2009). Cancer incidence and risk factors after solid organ transplantation. *International Journal of Cancer*.

[5] Gutierrez-Dalmau A., Campistol J. M. (2007). Immunosuppressive therapy and malignancy in organ transplant recipients. *Drugs*.

[6] Hanahan D., Weinberg R. A. (2011). Hallmarks of cancer: the next generation. *Cell*.

[7] Park J. M., Choi M.-G., Kim S. W. (2010). Increased incidence of colorectal malignancies in renal transplant recipients: a case control study. *American Journal of Transplantation*.

[8] Safaeian M., Robbins H., Berndt S., Lynch C., Fraumeni J., Engels E. (2016). Risk of colorectal cancer after solid organ transplantation in the United States. *American Journal of Transplantation*.

[9] Banerjea A., Ahmed S., Hands R. E. (2004). Colorectal cancers with microsatellite instability display mRNA expression signatures characteristic of increased immunogenicity. *Molecular Cancer*.

[10] Wang L., Cunningham J. M., Winters J. L. (2003). *BRAF* mutations in colon cancer are not likely attributable to defective DNA mismatch repair. *Cancer Research*.

[11] Venderbosch S., Nagtegaal I. D., Maughan T. S. (2014). Mismatch repair status and BRAF mutation status in metastatic colorectal cancer patients: a pooled analysis of the CAIRO, CAIRO2, COIN, and FOCUS studies. *Clinical Cancer Research*.

[12] Oliveira R. C., Tavares-Silva E., Abrantes A. M. (2020). De novo colorectal cancer after liver and kidney transplantation- Microenvironment disturbance. *Transplantation Reports*.

[13] Guba M., von Breitenbuch P., Steinbauer M. (2002). Rapamycin inhibits primary and metastatic tumor growth by antiangiogenesis: involvement of vascular endothelial growth factor. *Nature Medicine*.

[14] Maluccio M., Sharma V., Lagman M. (2003). Tacrolimus enhances transforming growth factor-beta1 expression and promotes tumor progression. *Transplantation*.

[15] Imao T., Ichimaru N., Takahara S. (2007). Risk factors for malignancy in Japanese renal transplant recipients. *Cancer*.

[16] Benson A. B., Venook A. P., Al-Hawary M. M. (2021). Colon Cancer, Version 2.2021, NCCN clinical practice guidelines in oncology. *Journal of the National Comprehensive Cancer Network*.

[17] André T., Shiu K.-K., Kim T. W. (2020). Pembrolizumab in microsatellite-instability-high advanced colorectal cancer. *The New England Journal of Medicine*.

[18] Abdel-Wahab N., Safa H., Abudayyeh A. (2019). Checkpoint inhibitor therapy for cancer in solid organ transplantation recipients: an institutional experience and a systematic review of the literature. *Journal for Immunotherapy of Cancer*.

[19] Papac R. J. (1996). Spontaneous regression of cancer. *Cancer Treatment Reviews*.

[20] Abdelrazeq A. S. (2007). Spontaneous regression of colorectal cancer: a review of cases from 1900 to 2005. *International Journal of Colorectal Disease*.

[21] Kloor M., von Knebel Doeberitz M. (2016). The immune biology of microsatellite-unstable cancer. *Trends Cancer*.

[22] Vogelstein B., Papadopoulos N., Velculescu V. E., Zhou S., Diaz L. A., Kinzler K. W. (2013). Cancer genome landscapes. *Science*.

[23] Waldman A. D., Fritz J. M., Lenardo M. J. (2020). A guide to cancer immunotherapy: from T cell basic science to clinical practice. *Nature Reviews. Immunology*.

[24] Chow M. T., Möller A., Smyth M. J. (2012). Inflammation and immune surveillance in cancer. *Seminars in Cancer Biology*.

[25] Kayhanian H., Barmpoutis P., Lakatos E. (2022). *Mutation Rate Evolution Drives Immune Escape in Mismatch Repair-Deficient Cancer*.

[26] Mizushima N. (2007). Autophagy: process and function. *Genes & Development*.

[27] Levine B., Kroemer G. (2008). Autophagy in the pathogenesis of disease. *Cell*.

